# LysX2 is a *Mycobacterium tuberculosis* membrane protein with an extracytoplasmic MprF-like domain

**DOI:** 10.1186/s12866-022-02493-2

**Published:** 2022-04-01

**Authors:** Francesca Boldrin, Laura Cioetto Mazzabò, Marie-Antoinette Lanéelle, Laura Rindi, Greta Segafreddo, Anne Lemassu, Gilles Etienne, Marta Conflitti, Mamadou Daffé, Alfredo Garzino Demo, Riccardo Manganelli, Hedia Marrakchi, Roberta Provvedi

**Affiliations:** 1 Department of Molecular Medicine, University of Padua, Padua, Italy; 2 Institut de Pharmacologie et de Biologie Structurale, IPBS, Université de Toulouse, CNRS, UPS, Toulouse, France; 3 Department of Translational Research, University of Pisa, Pisa, Italy; 4 Department of Microbiology and Immunology, School of Medicine, University of Maryland, Baltimore, Maryland USA; 5 Department of Biology, University of Padua, Padua, Italy

**Keywords:** MprF, Aminoacyl-phosphatidylglycerol synthase, Mycobacteria, Tuberculosis, LysX, Antimicrobial peptides, Acidic pH, Biofilm

## Abstract

**Background:**

Aminoacyl-phosphatidylglycerol (aaPG) synthases are bacterial enzymes that usually catalyze transfer of aminoacyl residues to the plasma membrane phospholipid phosphatidylglycerol (PG). The result is introduction of positive charges onto the cytoplasmic membrane, yielding reduced affinity towards cationic antimicrobial peptides, and increased resistance to acidic environments. Therefore, these enzymes represent an important defense mechanism for many pathogens, including *Staphylococcus aureus* and *Mycobacterium tuberculosis* (*Mtb*), which are known to encode for lysyl-(Lys)-PG synthase MprF and LysX, respectively. Here, we used a combination of bioinformatic, genetic and bacteriological methods to characterize a protein encoded by the *Mtb* genome, Rv1619, carrying a domain with high similarity to MprF-like domains, suggesting that this protein could be a new aaPG synthase family member. However, unlike homologous domains of MprF and LysX that are positioned in the cytoplasm, we predicted that the MprF-like domain in LysX2 is in the extracytoplasmic region.

**Results:**

Using genetic fusions to the *Escherichia coli* proteins PhoA and LacZ of LysX2, we confirmed this unique membrane topology, as well as LysX and MprF as benchmarks. Expression of *lysX2* in *Mycobacterium smegmatis* increased cell resistance to human β-defensin 2 and sodium nitrite, enhanced cell viability and delayed biofilm formation in acidic pH environment. Remarkably, MtLysX2 significantly reduced the negative charge on the bacterial surface upon exposure to an acidic environment. Additionally, we found LysX2 orthologues in major human pathogens and in rapid-growing mycobacteria frequently associated with human infections, but not in environmental and non-pathogenic mycobacteria.

**Conclusions:**

Overall, our data suggest that LysX2 is a prototype of a new class within the MprF-like protein family that likely enhances survival of the pathogenic species through its catalytic domain which is exposed to the extracytoplasmic side of the cell membrane and is required to decrease the negative charge on the bacterial surface through a yet uncharacterized mechanism.

**Supplementary Information:**

The online version contains supplementary material available at 10.1186/s12866-022-02493-2.

## Background

Aminoacyl-phosphatidylglycerol (aaPG) synthases are a large family of integral membrane proteins widely distributed among prokaryotes that catalyze biosynthesis of phosphatidylglycerol (PG) aminoacyl esters by using PG and aminoacyl-tRNA as substrates. As such, aaPG synthases modify anionic phospholipids with a cationic functionality and decrease the overall net negative charge of the plasma membrane leading to a reduced affinity for cationic antimicrobial peptides (CAMPs), antibiotics, and an increased resistance to acidic environments [1, 2]. This is particularly critical for pathogens such as *Staphylococcus aureus* or *Mycobacterium tuberculosis* (*Mtb*) as they face several acidic conditions during their infection process and counteract the action of CAMPs produced by the host defenses [1, 3–7]. In fact, the aaPG mechanism was first characterized for the multiple peptide resistance factor MprF from *Staphylococcus aureus* (SaMprF) shown to be a bifunctional protein with two separate domains, N-terminal hydrophobic domain and C-terminal hydrophilic domain [1, 8, 9]. In general, across the aaPG family, the N-terminal domain contains a variable number of transmembrane segments (TMSs) and functions as a flippase to translocate aaPGs from the inner leaflet to the outer leaflet of bacterial cytoplasmic membranes [1, 2, 10]. The hydrophilic C-terminal domain includes the active site that catalyzes formation of aaPG and is invariably located in the cytosol, which ensures access to aminoacyl-tRNA substrate. The hydrophilic domain is well-conserved across the aaPG synthases, whereas the hydrophobic domain is highly divergent [1]. Recently, cryo-electron microscopy (cryo-EM) structure of lysyl-PG (Lys-PG) synthase from the environmental rhizobia *Rhizobium tropici* (RtMprF) has been described [11], thus providing structural framework for aaPG synthase mechanism that was shown to involve tight coordination between aaPG synthesis and translocation across the membrane. In this structure, the flippase domain is tightly coupled to the synthase domain, which can assume multiple conformations. The flippase domain features two LysPG binding cavities, one facing the cytosol, which receives the Lys-PG from the synthase domain, and one facing the outer leaflet of the membrane, thus positioning Lys-PG for outer leaflet incorporation. Given the similarity between domain organization of RtMprF and other members of the family, it is expected that they all employ a similar mechanism.

In *Mtb*, production of Lys-PG depends on the presence of LysX (referred to as MtLysX in this work), an orthologue of SaMprF. MtLysX-mediated Lys-PG formation conferred resistance to CAMPs and low pH, and *lysX* deletion resulted in perturbed membrane potential, as well as defects in growth and intracellular replication [11–14]. Additionally, differential expression levels of *lysX* among various *Mtb* clinical isolates was shown to correlate with virulence, with more virulent strains expressing higher levels of MtLysX [15]. Yet, the presence of MtLysX is not strictly linked to virulence since MtLysX orthologues are encoded in most of the mycobacterial genomes, including environmental nonpathogenic species.

Here, we identified Rv1619 as an MtLysX orthologue present exclusively in pathogenic mycobacteria. Based on sequence similarity to MtLysX and SaMprF we annotated this protein as MtLysX2. Unexpectedly, our topology prediction results suggested that putative aaPG synthase of MtLysX2 is exposed to the extracytosolic side of the plasma membrane. We supported the validity of this prediction experimentally, thus showing that MtLysX2 may represent a unique member of aaPG synthase family. We showed that MtLysX2, when expressed in *M. smegmatis*, conferred an increased resistance to the human antimicrobial peptide β-defensin 2 (HBD-2) and to sodium nitrite. Remarkably, MtLysX2 reduced the negative charge on the bacterial surface upon exposure to an acidic environment, enhanced bacterial cell viability at lethal acidic pH and induced a slowdown in biofilm formation. Altogether, our data suggests a role of MtLysX2 in mycobacterial fitness.

## Results

### MtLysX2 is a membrane protein with a putative aaPG synthase domain

**Fig. 1:**
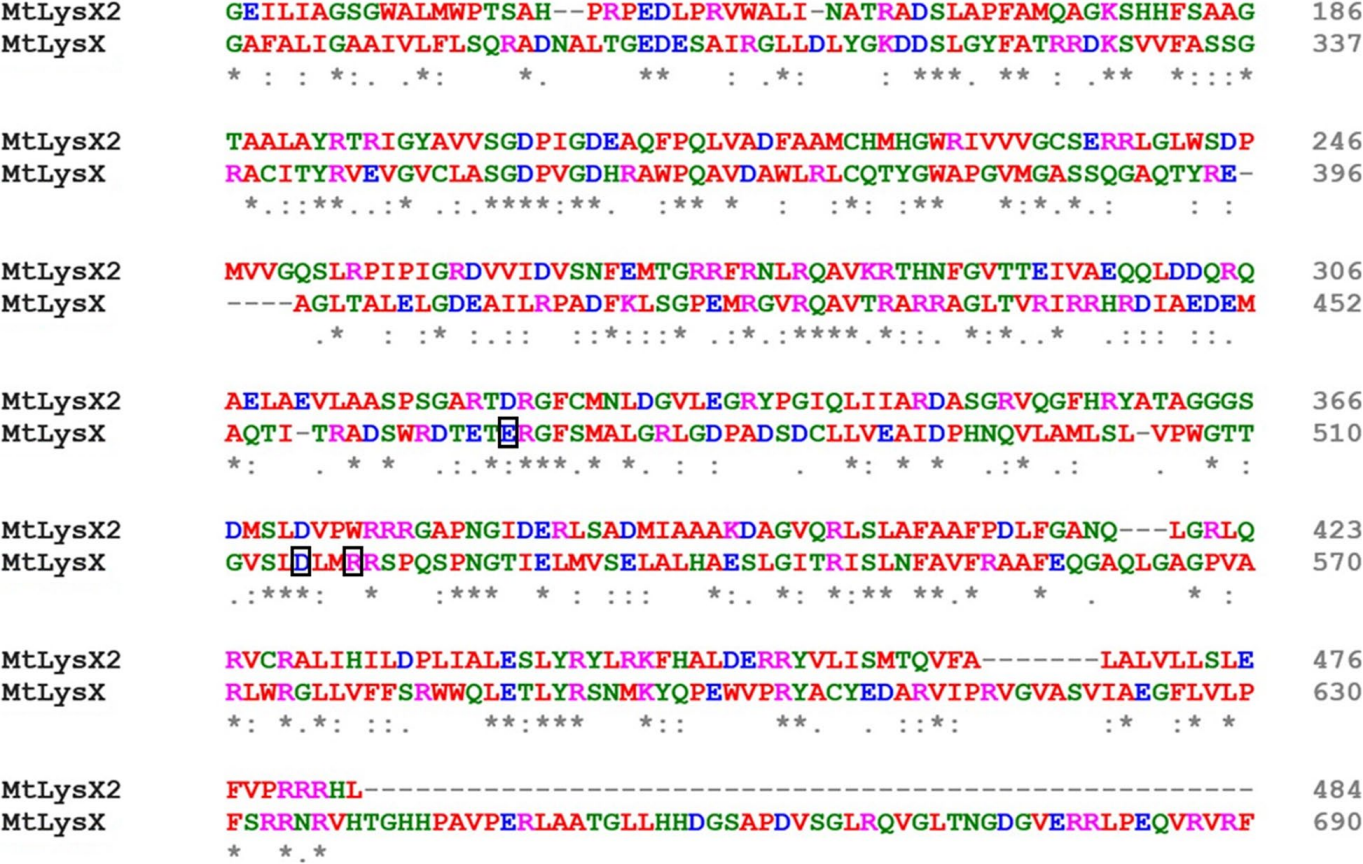
Sequence alignment of the catalytic domain of MtLysX and MtLysX2. Clustal Omega alignment of the MtLysX2 hydrophilic C-terminal region (355 amino acids) with the MtLysX homologous region involved in the Lys-PG biosynthesis. Amino acids shown to be required for the catalytic activity in SaMprF and conserved in MtLysX are boxed [8]. Asterisk: positions with a single and fully conserved residue; colon: positions with conservation between amino acid groups of strongly similar properties; period: positions with conservation between amino acid groups of similar properties

Rv1619 is annotated in the *Mtb* genome database as an integral membrane protein with unknown function. It includes a hydrophobic region that is predicted to form four helical transmembrane segments (TMSs) and a 343-amino acid hydrophilic domain sharing 27.5% sequence identity (56.6% similarity) with the hydrophilic Lys-PG synthase domain of MtLysX and 20% sequence identity (54.3% similarity) with SaMprF, both required for the synthesis of Lys-PGs in *Mtb*, and *S. aureus* respectively (Fig. 1). Furthermore, Rv1619 also shares a comparable sequence similarity with the Lys-PG synthase domain of MprF-like proteins from *Listeria monocytogenes* (21.3% identity, 53.1% similarity), *Pseudomonas aeruginosa* (24% identity, 47.5% similarity), *Bacillus licheniformis* (18.3% identity, 53% similarity), *Bacillus subtilis* (20.4% identity, 54.5% similarity), *Enterococcus faecalis* MprF2 (20.7% identity, 56.2% similarity), *Clostridium perfringens* MprF1 (21.9% identity, 56.3% similarity), *Clostridium perfringens* MprF2 (17.2% identity, 54% similarity) and *Rhizobium tropici* (21.4% identity, 53.8% similarity). As a comparison, MtLysX and SaMprF share 22.6% sequence identity (57% similarity), therefore suggesting that the similarity between Rv1619 and SaMprF is comparable to what has previously been seen in two established systems. Consequently, we assign a preliminary annotation of Rv1619 as MtLysX2.

### Membrane topology of MtLysX2

**Fig. 2:**
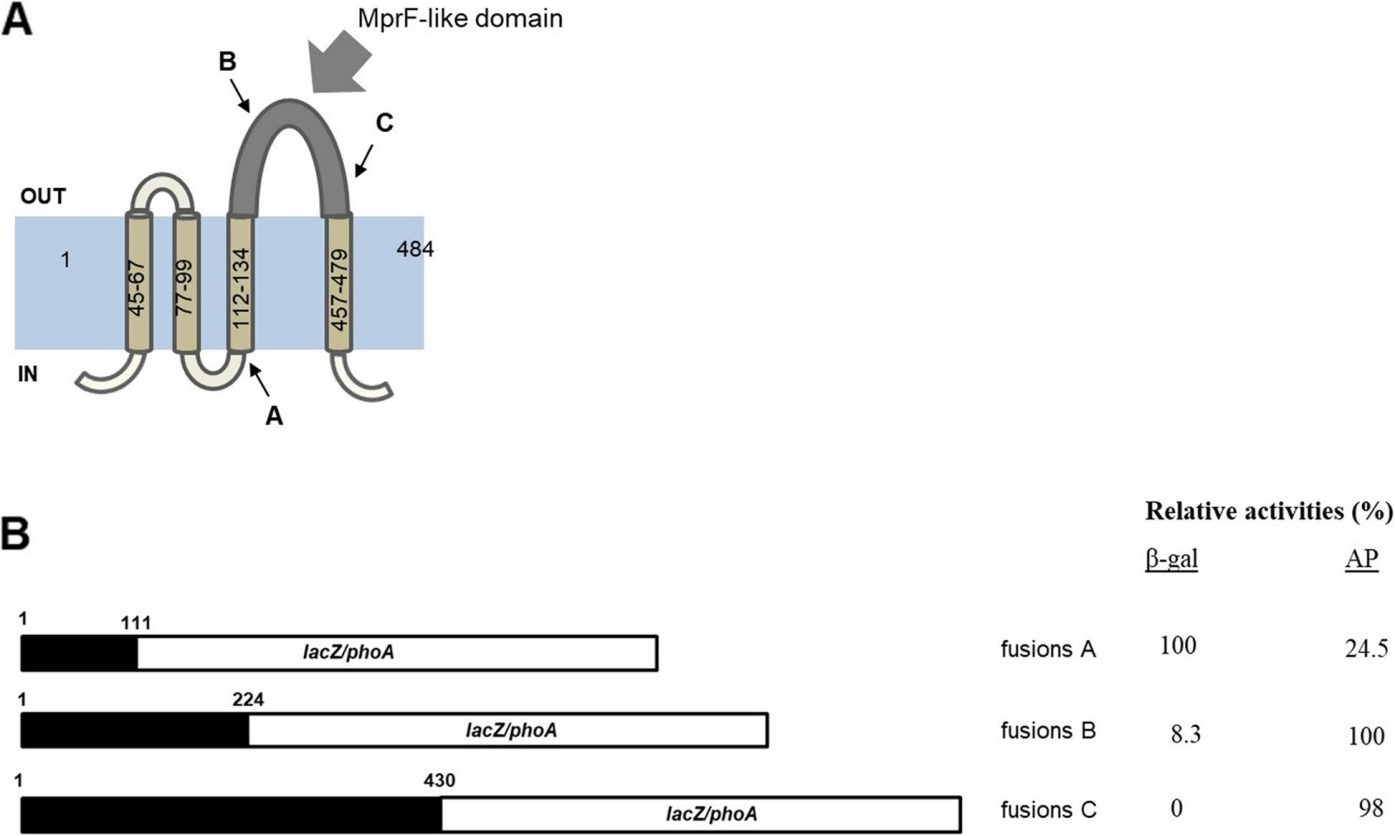
Membrane topology of MtLysX2. **A** Schematic representation of MtLysX2 (Rv1619) membrane topology as predicted by both HMMTOP and TMHMM. The MprF-like domain with sequence similarity to the Lys-PG synthase domain of SaMprF is highlighted in dark gray and pointed by a thick gray arrow. Thin arrows indicate the points chosen for fusions with either *lacZ* or *phoA*. **B** Schematic representation of the three different fragments of MtLysX2 (black) fused to either *phoA* or *lacZ* (white) and their relative activities, representative of two independent experiments. IN: inside the cytoplasm. OUT: outside the cytoplasm. The *phoA*/*lacZ* moiety is not drawn on scale as the two genes have a different length. Numbers indicate amino acid positions. β-galactosidase activities (β-gal) are normalized relative to that of fusion A. Alkaline phosphatase activities (AP) are normalized relative to that of fusion B

MtLysX2 is predicted to be an integral membrane protein that includes a hydrophobic region displaying 4 predicted helical transmembrane segments (TMSs) in addition to a MprF-like hydrophilic domain. To map the topology of MtLysX2, we used two well-established transmembrane protein topology prediction servers: HMMTOP (http://www.enzim.hu/hmmtop/) and TMHMM (https://services.healthtech.dtu.dk/service.php?TMHMM-2.0) [16, 17]. We benchmarked performance of the two servers using MtLysX and SaMprF. AaPG synthases are integral membrane proteins, with the synthase domain expected to be located on the cytosolic side of the membrane. Their hydrophilic Lys-PG synthase regions were predicted to localize on the cytosolic face of the membrane by both servers with the exception of SaMprF whose Lys-PG synthase regions was predicted to be in the cytoplasm only from one server, in agreement with that determined experimentally (See Supplementary Fig. 1, Additional file 1) [18]. Remarkably, prediction for the putative aaPG synthase domain of MtLysX2 by both servers indicated an unusual extracellular localization (Fig. 2 A). To investigate on which side of the plasma membrane the MprF-like domain of MtLysX2 was located, we used an assay performed in *Escherichia coli* based on gene fusions with PhoA or LacZ. This strategy has been successfully used to determine topology of membrane proteins from a large number of different bacterial species, including SaMprF [18–24]. It is based on the rationale that the alkaline phosphatase PhoA is active when translocated across the plasma membrane, while it is inactive if retained in the cytosol. Conversely, LacZ is active enzymatically only when localized in the cytosol and is inactivated when transported across the membrane [25–28]. Moreover, it was previously shown that MprF proteins can fold properly in the plasma membrane of *E. coli* upon heterologous expression and carry out their activity [3, 29, 30].

Three different 3’-deleted fragments of *Mtb lysX2*, four different deleted fragments of *Mtb lysX*, and two different 3’-deleted fragments of *S. aureus mprF* were cloned in frame with *phoA* and *lacZ*. Schematic representations of all the different fusions and their relative activities are shown in Fig. 2B and in Supplementary Fig. 1 (Additional file 1). Both LacZ fusions to SaMprF Lys-PG synthase were enzymatically active, while PhoA fusions were not, consistent with the hypothesis of cytoplasmic exposure of this domain (See Supplementary Fig. 1, Additional file 1). Similarly, LacZ fusions to MtLysX Lys-PG synthase and to MtLysU domain, (which was shown to be required for the production of Lys-PG [12]), were active, while the corresponding PhoA fusions were not active, consistent with cytoplasmic exposure of these two domains (See Supplementary Fig. 1, Additional file 1). However, the opposite was found for MtLysX2 MprF-like domain: in this case, only PhoA fusions were active, while LacZ fusions were not, suggesting that this domain is exposed to the periplasmic side of the membrane as predicted (Fig. 2). Taken together, our data suggest a peculiar topology of MtLysX2 with its MprF-like domain located on the periplasmic face of the cytoplasmic membrane.

### MtLysX2 is absent from environmental and non-pathogenic mycobacteria

To examine the distribution of MtLysX2 among other mycobacteria, we analyzed 54 mycobacterial species from across five distinct monophyletic clades within the genus *Mycobacterium: Tuberculosis-Simiae*, *Terrae*, *Triviale*, *Fortuitum-Vaccae*, and *Abscessus-Chelonae* [31]. Of these clades, the *Abscessus-Chelonae* and the *Fortuitum-Vaccae* are rapid-growing mycobacteria (RGM), whereas the *Tuberculosis-Simiae*, *Terrae* and *Triviale* are slow-growing mycobacteria (SGM). Our analysis included pathogenic, non-pathogenic, and environmental mycobacteria, to ensure broad coverage, and was done using BLASTP on taxonomic groups available in the NCBI database (https://blast.ncbi.nlm.nih.gov/). We identified MtLysX homologue in all of them except for a few species analyzed, suggesting that the presence of MtLysX does not differentiate between pathogenic and non-pathogenic mycobacteria (Tables 1 and 2). On the other hand, MtLysX2 was found in all species belonging to the *Tuberculosis-Simiae* clade of major human pathogens except *Mycobacterium leprae* and *Mycobacterium lepromatosis* (Table 2), and in the RGM species frequently associated with human infections such as *Mycobacterium fortuitum*, *Mycobacterium abscessus* and *Mycobacterium chelonae* (Table 1). Interestingly, we found that no MtLysX2 orthologues are present in *Mycobacterium smegmatis*, a saprophytic RGM belonging to the *Fortuitum-Vaccae* clade (Table 1). Taken together, our analysis suggests that MtLysX2 may represent a previously unannotated aaPG-like protein related to MtLysX that is distinctly present in pathogenic mycobacterial species.

**Table 1:**
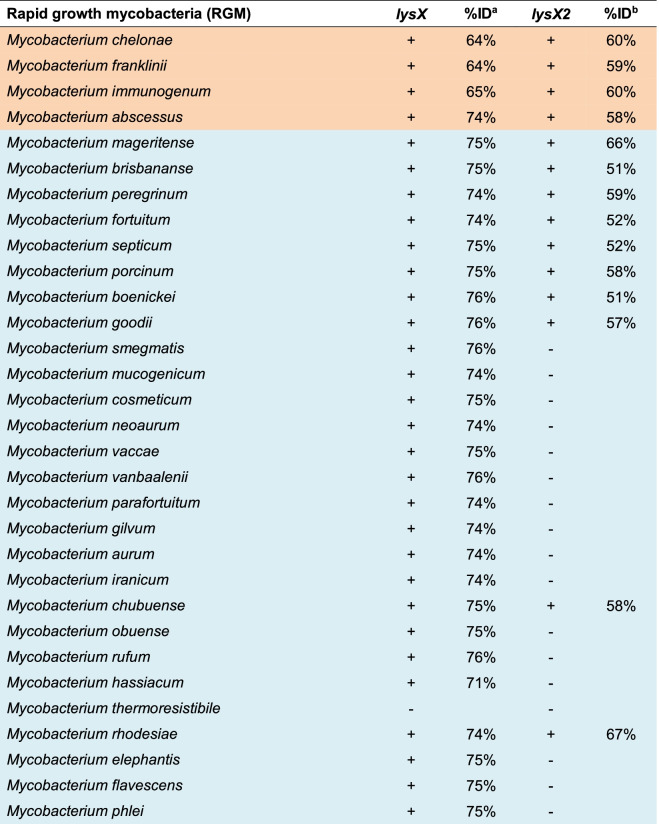
Distribution of LysX and LysX2 among different slow growth Mycobacterium species

Distribution of LysX and LysX2 among different slow growth Mycobacterium species

^a^%ID indicates the percentage of identical amino acids of the protein searched from Mycobacterium taxonomic groups (taxid) by BLASP and whose length spanned over 90% the amino acid sequence of LysX. ^b^ %ID indicates the percentage of identical amino acids of the protein searched from Mycobacterium taxonomic groups (taxid) by BLASP and whose length spanned over 80% the amino acid sequence of LysX2

**Table 2:**
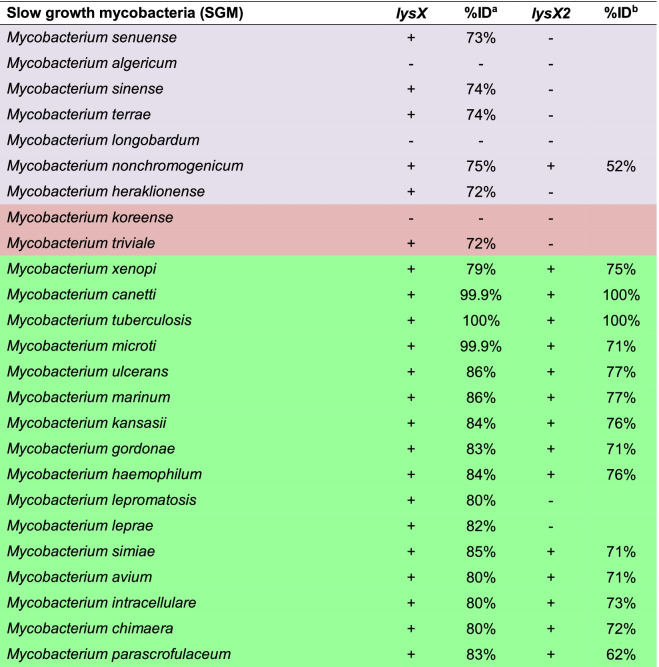
Distribution of LysX and LysX2 among different slow growth Mycobacterium species

Colors indicate the different clades: Terrae (lilac), Triviale (red), Tuberculosis-Simiae (green)

^a^%ID indicates the percentage of identical amino acids of the protein searched from Mycobacterium taxonomic groups (taxid) by BLASP and whose length spanned over 90% the amino acid sequence of LysX. ^b^ %ID indicates the percentage of identical amino acids of the protein searched from Mycobacterium taxonomic groups (taxid) by BLASP and whose length spanned over 80% the amino acid sequence of LysX2

### Heterologous expression of MtLysX2 in *M. smegmatis*

To examine whether MtLysX2 has unique functional roles given its unprecedented topology, we introduced a replicative plasmid bearing the *Mtb lysX2* gene and its putative native promoter into *M. smegmatis* strain mc^2^155. This strain has been successfully used as surrogate of *Mtb* [32–36], and does not encode endogenous MtLysX2. The plasmid we used is based on the replicon of pAL5000, which has an estimated copy number of three in *M. smegmatis* [37] theoretically guaranteeing a protein expression level close to the physiological expression occurring in *M. tuberculosis*. The recombinant strain was named MS322. In addition, a strain bearing the empty vector was also generated as control and was named MS321. As the activity of aaPG synthases, including SaMprF and MtLysX, increases resistance to CAMPs and acidic pH environments, we used these recombinant strains to explore whether MtLysX2 exhibited similar hallmarks.

### MtLysX2 confers increased resistance to the human β-defensin 2 and nitrosative stress

**Fig. 3:**
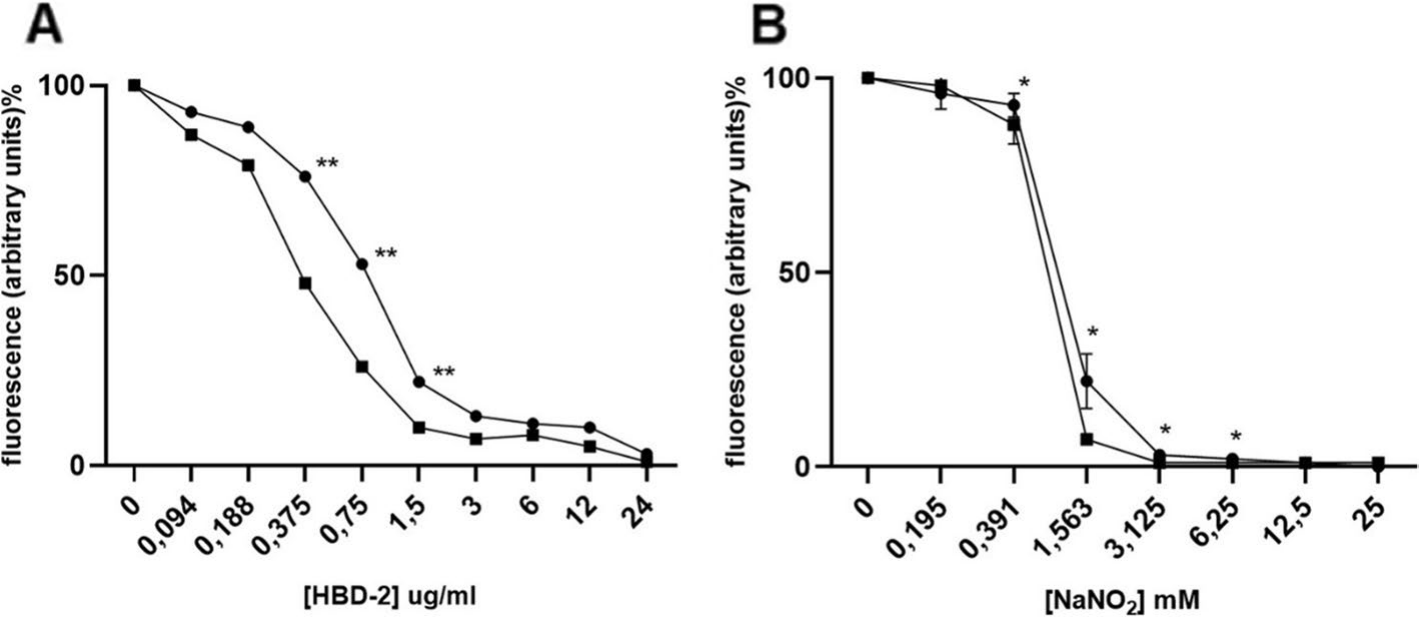
HBD-2 and NaNO_2_ minimum inhibitory concentration. **A** Minimum inhibitory concentration of HBD-2 in *M. smegmatis* mc^2^155::*MtlysX2* expressing MtLysX2 (MS322, dotted line) compared to the parental strain mc^2^155::pROL_Hyg (MS321, squared line). Error bars derive from two independent experiments. ** pValue ≤  0.005. **B** Minimum inhibitory concentration of NaNO_2_ in *M. smegmatis* mc^2^155::Mt_*lysX2* expressing MtLysX2 (MS322, dotted line) compared to the parental strain mc^2^155::pROL_Hyg (MS321, squared line). Error bars derive from two independent experiments. *p Value  ≤  0.05

To test whether the expression of MtLysX2 could be involved in increasing resistance to CAMPs, we analyzed sensitivity of MS322 and MS321 to two human CAMPs, defensins HNP-1 and HBD-2, using the Resazurin Microtiter Assay (REMA). This assay measures cell respiration through conversion of resazurin to resorufin and is commonly used to determine mycobacterial minimum inhibitory concentrations (MICs) to drugs [38]. As shown in Fig. 3 A, the *MtlysX2*-expressing strain MS322 exhibited an increased resistance to the human β-defensin 2 (HBD-2), as its MIC_90_ was two- to four-fold higher (3–6 µg/ml) than that of the control strain MS321 (1.5 µg/ml). However, the sensitivity of both strains to the human neutrophil peptide HNP-1 was unchanged (See Supplementary Fig. 2, Additional file 1). Our data indicate that MtLysX2 is functional and confers partial resistance to CAMPs when expressed in *M. smegmatis*.

Recently, it has been shown that LysX from *Mycobacterium avium hominissuis* influenced tolerance toward oxidative and nitrosative stress [39], which are two stress conditions encountered by *Mtb* during macrophage infection. Similarly, also MtLysX exhibited moderately increased sensitivity to these two stresses [14]. Therefore, we also tested whether MtLysX2 was required to mediate mycobacterial adaptation to nitrosative and oxidative stress. As for HBD-2 and HNP-1, a REMA assay was performed to analyze tolerance to sodium nitrite, which is well known to induce nitrosative stress at pH 5.5 [40]. The MIC_90_ to MS322 was two-fold higher (3.125 mM) compared to that of the control strain MS321 (1.56 mM). Interestingly, a statistically significant difference in the survival was observed starting from 0.39 mM, a concentration that mimic physiologically relevant conditions (Fig. 3B) [40]. However, a quantitative assessment of the bacterial survival of MS321 and MS322 following exposure to 5 mM H_2_O_2_ exhibited no significant difference (See Supplementary Fig. 3, Additional file 1).

### MtLysX2 confers increased resistance to lethal acidic pH and delays biofilm formation

**Fig. 4:**
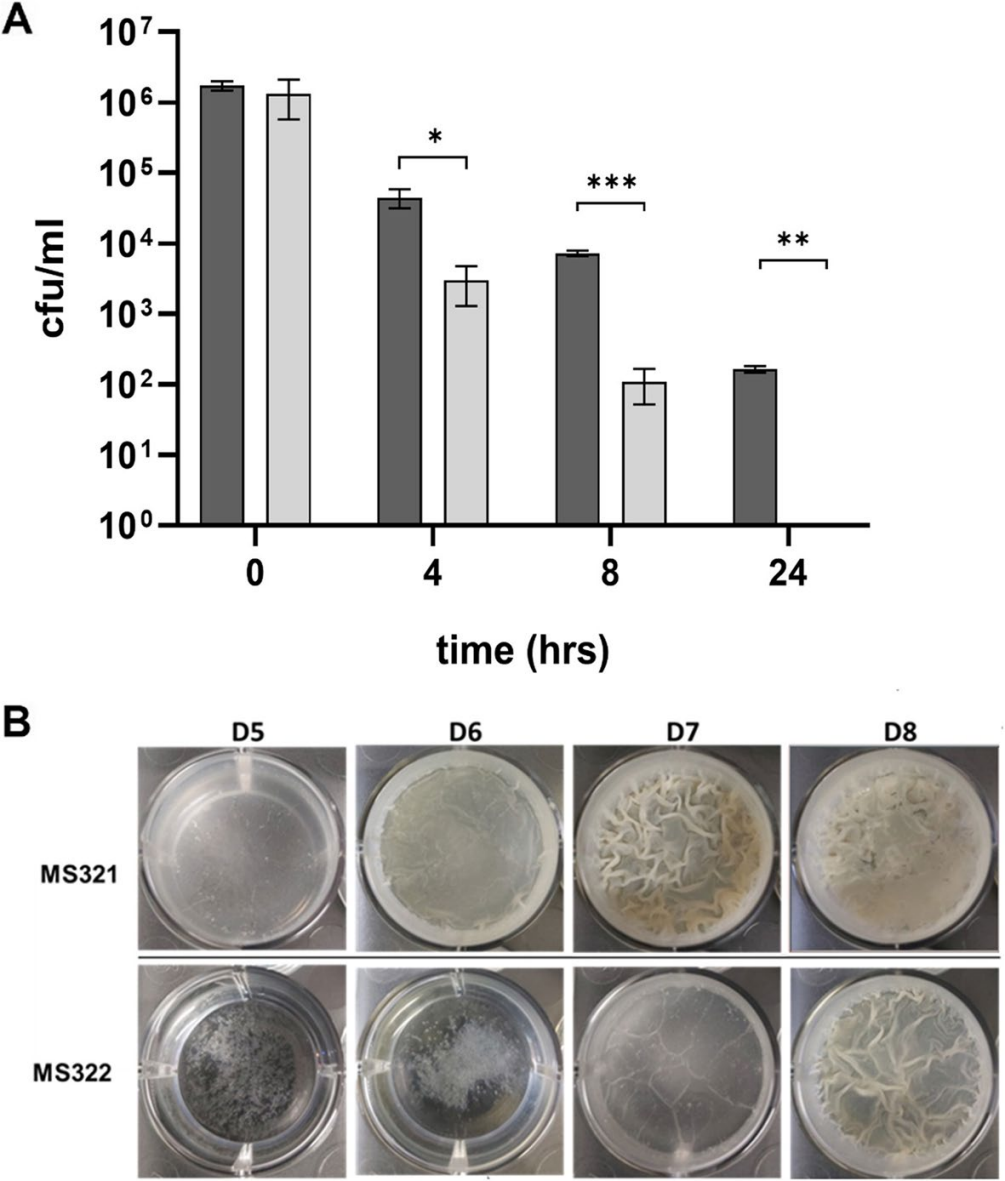
MtLysX2 expression increases resistance to lethal acidic pH and delays biofilm formation. **A** Killing assay of *M. smegmatis* mc^2^155::*MtlysX2* (MS322, dark gray) and mc^2^155::pROL_Hyg (MS321, light gray) at pH 4.5. Strains were grown for 24 h in Sauton medium at pH 5.5 before being transferred into the same medium at pH 4.5. *p = 0.48; **p = 0.01, ***p = 0.0045. **B** Biofilm formation in Sauton medium pH 5.5. Bacteria, grown to stationary phase were diluted 1:100 in Sauton medium pH 5.5. 4 ml of the suspension were than inoculated in a 24-well plate and incubated at 37 °C. Pictures were taken after 5, 6, 7, and 8 days (**D**). The experiment was repeated twice in triplicate obtaining comparable results

Next, we examined whether expression of MtLysX2 in MS322 could enhance cell viability under lethal acidic conditions. Indeed, the viability of MS322 after 8 h of incubation at pH 4.5 was 40-times higher than that of MS321, and no viable cells could be detected after 24 h exposure at pH 4.5 in the absence of MtLysX2 (Fig. 4 A). Interestingly, such a difference in the survival rate of both strains could be observed only when cells were transferred to pH 4.5 after 24 h pre-growth in Sauton medium at moderately acidic conditions of pH 5.5. No difference in the survival rate at pH 4.5 was observed when cells were pre-grown in Sauton medium at pH 6.8 or in 7H9 medium at either pH (See Supplementary Fig. 4, Additional file 1). This phenotype was not dependent on a putative differential expression level of *MtlysX2* at the tested pH conditions as the relative amount of its mRNA was the same after 24 h of cultivation at either pH 5.5 or 6.8 (1.03 ± 0.28). This suggests that exposure to mild acidic pH could have an effect either on the activity of MtLysX2 or on its function.

Then we explored the ability of MtLysX2 to interfere with biofilm formation in *M. smegmatis*, since MprF from *Listeria monocytogenes* and *Enterococcus faecalis* were shown to delay the formation of biofilms [41, 42]. MS322 and MS321 were cultivated in Sauton medium at pH 6.8 or 5.5 in 12-well plates. As shown in Fig. 4B, MS322 biofilm formation was delayed at pH 5.5 compared to the control strain MS321, suggesting that in these conditions the presence of MtLysX2 is detrimental to biofilm development, even though not interfering with dispersed growth (See Supplementary Fig. 5, Additional file 1). Notably, this phenotype was not detectable when biofilms were grown at pH 6.8 (See Supplementary Fig. 6, Additional file 1).

### Zeta potential analysis

**Fig. 5:**
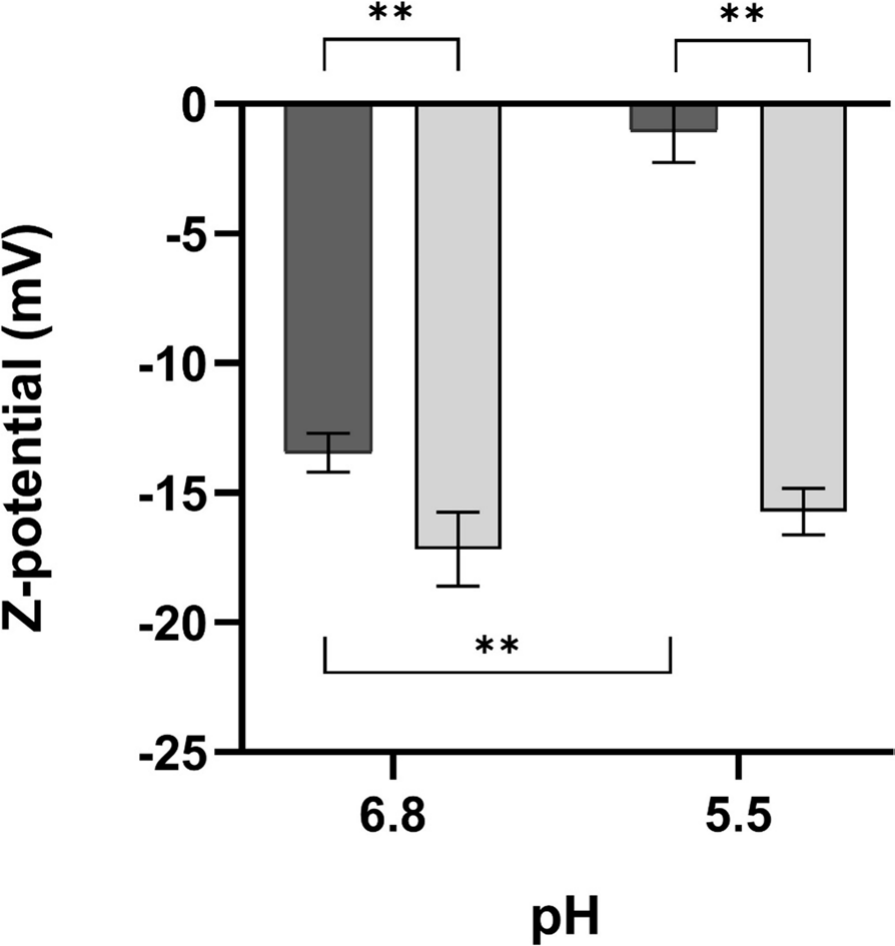
Zeta potential of *M. smegmatis* expressing MtLysX2. Zeta potential of *M. smegmatis* mc^2^155::*MtlysX2* (MS322, dark gray) and mc^2^155::pROL_Hyg (MS321, light gray) after growing for 24 h in Sauton at pH 6.8 (left) or 5.5 (right). **p < 0.005

Taken together, our experiments using a strain of *M. smegmatis* expressing MtLysX2 suggest that this protein could interfere with bacterial surface charge. To verify whether the presence of MtLysX2 induced a charge alteration on the bacterial surface, we analyzed the Zeta potential of MS321 and MS322 grown in Sauton medium at either pH 5.5 or pH 6.8. As shown in Fig. 5, a reduced negative charge was detected on the surface of the strain expressing MtLysX2 regardless of pH. Remarkably, while the surface charge difference between MS321 and MS322 was about 4 mV when bacterial cells were grown in Sauton medium at pH 6.8, a 15 mV difference was observed after growth at pH 5.5.

### Lipid analysis of *M. smegmatis* expressing MtLysX2

**Fig. 6:**
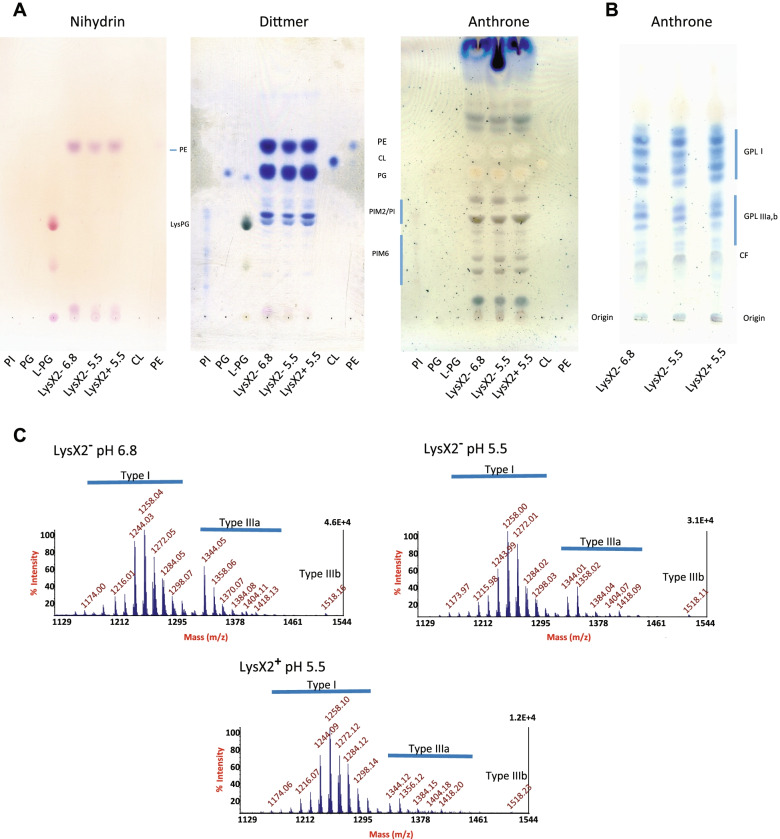
1D-Thin layer Chromatography (TLC) and MALDI-TOF mass spectrometry analysis of total lipid extracts from *M. smegmatis* MS321 (mc^2^155::pROL_Hyg ) and MS322 (mc^2^155::*MtlysX2*) grown at pH 6.8 and pH 5.5. **A** Revelations of the same plate were performed using different dyes: ninhydrin for free amino groups, Dittmer reagent for phosphate esters and anthrone for glycolipids. Solvent: CHCl_3_/CH_3_OH/H_2_O (60:35:8 v/v/v). Standards: LPG (Lysyl-PG), PI (phosphatidyl inositol), PG (phosphatidyl glycerol), CL (cardiolipin), PE (phosphatidyl ethanolamine), PIM (phosphatidylinositol mannosides). **B** Glycopeptidolipids (GPLs) were analyzed by employing CHCl_3_/CH_3_OH (90:10 v/v) as running solvent. The GPLs were detected with anthrone reagent and appear as blue spots (CF : cord factor). (C) MALDI-TOF mass spectra of the crude GPL fractions (GPL I : apolar diglycosylated GPLs ; GPL IIIa : succinylated diglycosylated GPLs ; GPL IIIb : succinylated triglycosylated GPLs)

In order to further investigate possible involvement of MtLysX2 in cell lipid modulation, we cultured MS321 and MS322 strains at pH 5.5 and pH 6.8 and analyzed their lipid content. First, we performed thin-layer chromatography (TLC) to analyze complex lipids using appropriate reagents, as described in the Methods section. We identified many phospholipid constituents of *M. smegmatis*, mainly the anionic phospholipids phosphatidyl inositol (PI), phosphatidyl glycerol (PG), cardiolipin (CL) and phosphatidyl inositol mannosides (PIM) (Fig. 6 A). Phosphatidylethanolamine (PE) was the only detected ninhydrin-positive phospholipid, while neither Lys-PG nor other amino acyl phospholipid were detected (Fig. 6B). Moreover, the search for potential Lys-PG from *M. tuberculosis* by MALDI-TOF mass spectrometry, exhibiting a molecular ion at m/z 681 (M-H) as isolated by Maloney et al. after growth under labeled lysine [12], was negative. Very recently, it was reported that the *lysX* gene of *Mycobacterium avium hominissuis* interferes with the surface glycopeptidolipids GPL expression profiles [39]. We therefore performed the comparative analysis of the GPL patterns of *M. smegmatis* MS321 and MS322 by TLC and MALDI-TOF mass spectrometry, as described [43]. TLC results showed no obvious difference under these experimental conditions (Fig. 6B). On the other hand, mass spectrometry analysis suggested that the ratio of polar type IIIa,b GPLs/apolar type I GPLs is lower when strain MS321 was cultured at pH 5.5, and decreased again when LysX2 was expressed (Fig. 6 C). Collectively, our lipid analysis of the MtLysX2-expressing strain, as well as that of the control strain, failed to yield clear indications about the production of Lys-PG or other aaPG. Therefore, additional experiments using either alternative lipid analysis methodologies and/or MtLysX2 producer strains will be needed to further examine the role of this protein in the process of aaPG synthesis.

## Discussion

MprF-like enzymes are widely represented among prokaryotes since several bacterial species, including firmicutes, actinobacteria and several proteobacteria, use them to produce aminoacyl esters of phosphatidylglycerol (aaPG) [1]. The result of this activity is a decreased net negative charge, and increased resistance to different environmental stress conditions such as low pH or exposure to CAMPs. In *Mtb*, these properties enable the pathogen to persist and survive within phagolysosomes of activated macrophages. MtLysX has been shown as critically important for supporting survival, virulence and conferring resistance to CAMPs and low pH in *Mtb* by producing Lys-PG [12, 13]. However, as MtLysX orthologues are encoded in most of the mycobacterial genomes, including environmental nonpathogenic species, this enzyme seems to be of general importance for the fitness of mycobacteria and not a distinct feature of pathogenic mycobacteria.

In this study, we identified a new *Mtb* gene encoding an additional MprF-like domain protein (Rv1619), which we named LysX2 (MtLysX2). Among 54 mycobacterial species analyzed, MtLysX2 orthologues were conserved only in the slow-growing mycobacteria including the major human pathogens, such as *Mtb*, and in rapid-growing mycobacteria frequently associated with human infections, for example *M. fortuitum*, *M. abscessus* and *M. chelonae*. Two exceptions were *M. leprae* and *M. lepromatosis*, whose genomes have undergone massive gene decay and reductive evolution, likely resulting in the loss of *lysX2*, among others [44, 45]. Interestingly, LysX2 was absent in most of the environmental mycobacteria that are not pathogenic or rarely associated with infections in humans such as the saprophytic *M. smegmatis*. This distinct species distribution suggests a more specialized role of this protein in pathogenesis compared to MtLysX.

Another key difference we observed between MtLysX and MtLysX2 is in their topology. Whereas the Lys-PG synthase domain of MtLysX and SaMprF is exposed to the cytosol, consistent with its role in transferring a lysyl residue from a tRNA^Lys^ to PG, our data suggest that the MprF-like domain of MtLysX2 is periplasmic. The reliability of the topology determined in our experimental model was further supported by the confirmation that also the Lys-tRNA synthase domain (LysU), which is fused to the Lys-PG synthase of MtLysX, showed a cytoplasmic localization consistent with the proposal that a dedicated Lys-tRNA synthase supplying a donor Lys-tRNA might lead to a more efficient biosynthesis of Lys-PGs. Indeed, the LysU domain is essential for the biosynthesis of Lys-PGs in *Mtb* [12]. This represents an unprecedented topology among aaPG synthase family members and suggests a peculiar mechanism of action.

A recent study based on the analysis of cryo-electron microscopy structures of MprF from *R. tropici* (RtMprF) revealed how MprF proteins operate [11]. Like SaMprF, RtMprF is characterized by a C-terminal hydrophilic domain with Lys-PG synthase activity and a large N-terminal hydrophobic domain carrying the flippase activity required to translocate Lys-PG from the inner leaflet to the outer leaflet of the cytoplasmic membrane. Song et al. [11] showed that the synthase domain undergoes conformational changes allowing its interaction with the hydrophobic domain to approach the membrane surface and acquire efficiently the lipid substrate (PG). This finding fully supported previous studies showing that the catalytic domain plus six out of the fourteen TMSs of SaMprF were sufficient for full-level Lys-PG biosynthesis, however, a further truncation abolished Lys-PG production, indicating that at least six TMSs were required for maintaining a functional enzyme [8]. Of note, the N-terminal hydrophobic domain of MtLysX has six TMSs, suggesting that MprF-like proteins require a minimum of six TMSs to fulfill their activity. Interestingly, the N-terminal hydrophobic domain of MtLysX2 has only three TMSs. This leads us to speculate that the catalytic domain of MtLysX2 may not undergo the conformational changes required by MprF proteins to approach PGs, suggesting that the substrate of MtLysX2 could be different.

MtLysX2 conferred a strikingly increased resistance to the killing caused by an acidic environment whose pH was as low as pH 4.5, a condition encountered inside phagolysosomes upon macrophage activation. Additionally, we observed that the presence of MtLysX2 strongly delayed the onset of biofilm development and maturation at pH 5.5, but not at pH 6.8. The biofilm phenotype is consistent with observation that *Listeria monocytogenes* and *Enterococcus faecalis mprF* deletion mutants produced significantly more biofilm than the wild type [41, 42]. Given the importance of cell surface contacts in biofilm formation, these phenotypes may be related to a modification of net charge on the bacterial cell surface. Indeed, the inspection of the Zeta potential of *M. smegmatis* expressing (or not) MtLysX2 indicated that MtLysX2 induced a reduction in the net negative charge on the cell surface, a phenomenon that was significantly enhanced at pH 5.5. Taken together, our studies under different pH conditions suggest that exposure to mild acidic pH does not change MtLysX2 expression, but rather has an effect on either the activity of MtLysX2 or on its function that results in MtLysX2-dependent modifications of the bacterial surface.

Given that MprF-like proteins catalyze formation of aaPGs, and thus change bacterial cell membrane lipid composition, we also analyzed the lipid profile of *M. smegmatis* expressing MtLysX2 under different pH conditions. Unexpectedly and despite our efforts, no difference was observed in phospholipid composition of MtLysX2-expressing and control strains. We were also unable to detect any Lys-PGs, which is not surprising as *M. smegmatis* does not have Lys-PG as constituent of its phospholipid pool. A possible reason for not detecting any changes in phospholipids could stem from low levels of *MtlysX2* expression resulting in lipid composition changes below our detection limits. On the other hand, mass spectrometry analysis suggested that the ratio polar type IIIa,b GPLs/apolar type I GPLs was lower when strain MS321 was cultured at pH 5.5, and decreased further when LysX2 was expressed. Although GPLs are absent in *Mtb*, these findings suggest that MtLysX2 could modulate the surface components whose expression may depend on environmental conditions.

In addition to low pH, *Mtb* has to face additional hostile environments generated by the host defense mechanisms during the infectious process, such as production of CAMPs or oxidative and nitrosative stress generated by reactive oxygen species (ROS) and reactive nitrogen species (RNS). Our initial evaluation of the MtLysX2-dependent phenotypes demonstrated that the presence of MtLysX2 conferred resistance to both the human defensin HBD-2, and sodium nitrite (a nitrosative stress inducer at pH 5.5) but did not enhance bacterial survival upon exposure to the human neutrophil peptide HNP-1 or hydrogen peroxide. The reduced negative charge observed on the surface of the bacterial strain expressing MtLysx2 was much more significant after growing the bacteria at pH 5,5 instead of pH 6,8. This condition, however, could not be used to test the sensitivity to HBD-2 and HNP-1 because a pH as low as 5.5 interfered with the CAMPs’ activity. It is possible that the surface net negative charge reduction however observed upon expression of MtLysx2, regardless of pH, can increase resistance to HBD-2 but not to HNP-1 because of the distinct structure and net positive charge of these two CAMPs (the former has a higher positive charge than the latter) which can employ a differential action against which MtLysX2 may or may not confer effective resistance [46].

We cannot exclude the possibility that the unique topology of MtLysX2 endows it with a different, still unknown mechanism and function. This leads us to hypothesize that after a duplication event of the gene encoding LysX, in slow growing mycobacteria, *lysX2* has gone through rearrangement events leading to the periplasmic exposure of its MprF-like domain. The significantly reduced negative charge observed in the strain expressing MtLysX2 after growth at pH 5.5 could be related to amino acylation of a component of the cell envelope catalyzed by the periplasmic domain of MtLysX2 during its transport across the cytoplasmic membrane. Cytoplasmic aa-tRNAs are the donor substrate of MprF-like proteins. It has been shown that the host immune response can be modulated by the action of microbial tRNA fragments secreted or delivered into host cells through extracellular vesicles [47–50]. In *Mtb*, extracellular RNA fragments derived by tRNA and rRNA were found to stably accumulate in the culture filtrate and play a role in inducing apoptosis in human monocytes. Among these extracellular mycobacterial RNAs, also tRNA^Lys^ derived fragments were detected [51]. Roy and Ibba demonstrated that truncated lysyl- or alanyl-tRNAs consisting of only the acceptor and T stems can also be used as substrate by MprF [2]. Therefore, we cannot exclude that extracellular tRNA derived fragments secreted by *Mtb* can accumulate in the periplasmic space and be a suitable substrate for MtLysX2.

### Conclusions

MtLysX2 is an MprF-like protein with likely role in enhancing survival of the mycobacterial pathogenic phenotypes as MtLysX2 orthologues were found in major human pathogens and in rapid-growing mycobacteria frequently associated with human infections, but not in environmental and non-pathogenic mycobacteria. We presented evidence that MtLysX2 considerably reduces the overall net negative charge on bacterial surface when bacteria are exposed to an acidic environment. Moreover, our findings suggest that its catalytic domain is exposed to the extracytoplasmic side of the cell membrane, highlighting a significant peculiarity of MtLysX2 compared to other family members that have strict requirement for cytoplasmic localization of MprF-like domain. Overall, our data show that MtLysX2 could be a prototype of a new class within the MprF-like protein family exerting its function through a novel still unknown mechanism which is important for modulating cell surface and consequently the bacterial fitness.

## Methods

### Bacterial strains, media, and growth conditions

All strains used in this study are listed in Table 3. *E. coli* strains were grown at 37 °C in Luria-Bertani (LB) broth or on LB agar plates. *M. smegmatis* strains were grown at 37 °C in Middlebrook 7H9 broth (Difco, Franklin Lakes, NJ, USA) supplemented with 0.2% glycerol and 0.05% Tween-80 or in Sauton medium (3.67 mM K_2_HPO_4_, 4 mM MgSO_4_, 30 mM L-Asparagine, 0.18 mM Ferric Ammonium Citrate, 23.8 mM Citric Acid, 4 mM Glycerol, 0.1 ml 1% Zinc Sulfate, 0.05% Tween-80). The media pH was then adjusted to 6.8, 5.5 or 4.5 with HCl (7H9) or NaOH (Sauton). To check cell viability, 7H10 agar plates (Difco) supplemented with 0.2% glycerol and 0.05% Tween-80 were used. When required, antibiotics were added to the media at the following concentrations: kanamycin (Km; Sigma) 50 µg ml^− 1^ (*E. coli*) or 20 µg ml^− 1^ (*M. smegmatis*), hygromycin (Hyg, Thermo Fisher Scientific) 150 µg ml^− 1^ (*E. coli*) or 50 µg ml^− 1^ (*M. smegmatis*), ampicillin (Am; Sigma) 100 µg ml^− 1^ (*E. coli*). Human β-defensin HBD-2 and α-defensin HNP-1 (kindly provided by Prof. Wuyuan Lu, University of Maryland, School of Medicine, Baltimore, USA) were dissolved in sterile milliQ water for stock preparations.

**Table 3:** List of strains

Strains	Relevant genotype and/or characteristics	Source or reference
***Escherichia coli***		
DH5α	F- *end A1 glnV44 thi- 1 recA1 relA1 gyrA96 deoR nupG purB20* φ80d*lacZ*ΔM15 Δ(*lacZYA-argF*)U169, hsdR17(*r*_*K*_^−^*m*_*K*_^+^), λ^−^	Laboratory stock
JM109	endA1 glnV44 thi-1 relA1 gyrA96 recA1 mcrB^+^ Δ(lac-proAB) e14- [F’ traD36 proAB^+^ lacI^q^ lacZΔM15] hsdR17(r_K_^−^m_K_^+^)	Laboratory stock
***M. smegmatis***		
mc^2^155	parental strain	Laboratory stock
MS321	mc^2^155 with pROL-HYG	This work
MS322	mc^2^155 with pROL-HYG::*Mt_lysX2*	This work

### DNA manipulation

All recombinant DNA techniques were performed according to standard procedures using *E. coli* DH5α or JM109 as the initial host. DNA restriction and modifying enzymes were obtained from New England Biolabs and used according to the manufacturer’s recommendations. All plasmids and primers used in this work are listed in Table 4 and additional file 1.

**Table 4:** Bacterial plasmids

Bacterial plasmids^a^	Relevant characteristics	Source or reference
pCR-Blunt II-TOPO	*ccdB* lethal gene ORF	Invitrogen Life Technology
pSM316	Integrative vector	[55]
pUCCMPHOA	Integrative vector for in frame fusions to *phoA* gene of *E. coli*	[52]
pJF751	Integrative vector for in frame fusions to *lacZ* gene of *E. coli*	[24]
pFRA30 (A)	*Mt_lysX2* (A111) cloned in pUCCMPHOA	This work
pFRA29 (A)	*Mt_lysX2* (A111) cloned in pJF751	This work
pFRA31 (B)	*Mt_lysX2* (H224) cloned in pUCCMPHOA	This work
pFRA32 (B)	*Mt_lysX2* (H224) cloned in pJF751	This work
pFRA35 (C)	*Mt_lysX2* (L430) cloned in pUCCMPHOA	This work
pFRA36 (C)	*Mt_lysX2* (L430) cloned in pJF751	This work
pFRA144(D)	*Mt_lysX* (P259) cloned in pUCCMPHOA	This work
pFRA162 (D)	*Mt_lysX* (P259) cloned in pJF751	This work
pFRA159 (E)	*Mt_lysX* (L488) cloned in pUCCMPHOA	This work
pFRA156 (E)	*Mt_lysX* (L488) cloned in pJF751	This work
pFRA160(F)	*Mt_lysX* (S592) cloned in pUCCMPHOA	This work
pFRA157 (F)	*Mt_lysX* (S592) cloned in pJF751	This work
pFRA161 (G)	*Mt_lysX* (Q699) cloned in pUCCMPHOA	This work
pFRA158 (G)	*Mt_lysX* (Q699) cloned in pJF751	This work
pFRA176 (H)	*Sa_mprF*(S521) cloned in pUCCMPHOA	This work
pFRA177 (H)	*Sa_mprF*(S521) cloned in pJF751	This work
pFRA178 (I)	*Sa_mprF*(F615) cloned in pUCCMPHOA	This work
pFRA179 (I)	*Sa_mprF*(F615) cloned in pJF751	This work
pROL-HYG	replicative plasmid	[37]
pROL-HYG::*Mt_lysX2*	*Mt_lysX2* + 543 bp upstream cloned inpROL-HYG	This work

^a^The letters in parentheses designate the fusion proteins encoded by the plasmids. ^b^The last aminoacyl residue included in the fusion is given in parentheses

### Construction of plasmids with *phoA* and *lacZ* fusions

Fusions of *Mt_lysX2*, *Mt_lysX* and *Sa_mprf* to either *phoA* or *lacZ* were generated by cloning fragments, previously amplified by PCR, in frame with either the *phoA* reporter gene of plasmid pUCCMPHOA [52] or the *lacZ* reporter gene of plasmid pJF751 [24]. H37Rv genomic DNA was used as template to clone fragments of *Mt_lysX2* and *Mt_lysX*, whereas *S. aureus* SA113 genomic DNA (Leibniz Institute DSMZ German collection of microorganisms and cell cultures) was used as template to clone fragments of *Sa_mprF*. Hybrid protein-coding genes with fusions between different amino-terminal regions of MtLysX2, MtLysX or SaMprF and the 15th residue of PhoA, were constructed by PCR primers that placed sections of the proteins in frame with PhoA. All the fragments amplified by PCR were first cloned in pCR-Blunt II-TOPO and then subcloned in pUCCMPHOA. Similarly, constructs containing the same fragments of MtLysX2, MtLysX or SaMprF fused to the 8th codon of *lacZ* were generated in pJF751. Restriction analysis and DNA sequencing confirmed the junction of each fusion construct. To generate pFRA30 (*Mt_lysX2::phoA* fusion A), pFRA29 (*Mt_lysX2::lacZ* fusion A), pFRA144 (*Mt_lysX::phoA* fusion D) and pFRA162 (*Mt_lysX::lacZ* fusion D), a sequence recognized by ribosomes (RBS) and by the NdeI restriction enzyme were also included in the forward primer before the first codon (Table 4 and Supplementary Table 1 in Additional file 1). These vectors were then used to replace the cloned fragment fused to either *phoA* or *lacZ* with all the other fragments from *Mt_lysX*, *Mt_lysX2* and *Sa_mprF*. When required, additional nucleotides were introduced in the reverse primers to maintain the reading frame. Restriction enzymes used for cloning are indicated in Supplementary Table 1 (Additional file 1).

### PhoA and LacZ activity assays with recombinant strains

The *E. coli* host strain used for the alkaline phosphatase (PhoA) and β-galactosidase (LacZ) assay were DH5α and JM109 respectively. We used DH5α as a host for PhoA assays since no PhoA activity background was detected in this strain. Recombinant strains transformed with truncated *Mt_lysX2, Mt_lysX*, and *Sa_mprF* versions fused to either *phoA* or *lacZ* of pUCCMPHOA or pJF751 respectively, were grown overnight in LB medium containing ampicillin. Each overnight culture was diluted 1:100 with fresh medium and grown up to OD_600_ of 0.5. Subsequently, expression of the PhoA or LacZ fusions was induced with 1 mM IPTG (Sigma-Aldrich) for 1 h. PhoA or LacZ activity were measured as follows. For the alkaline phosphatase assay, 1 ml of *E. coli* DH5α cells bearing the recombinant plasmids were harvested and resuspended in 1 ml of buffer A (1 M Tris-HCl pH 8, 0.1mM ZnCl2, 1mM iodoacetamide). Cells were permeabilized by the addition of 10 µl of toluene to allow entrance of the PhoA substrate p-nitrophenyl phosphate into the cells and incubated on ice for 30 min. Then 100 µl of p-nitrophenyl phosphate 0.4% (Sigma- Aldrich) was added and the samples were incubated at 37 °C for 30 min; the reaction was stopped with 120 µl of 1 M KH_2_PO_4_. After centrifugation, A_420_ of supernatants were determined. To evaluate the LacZ activity, 1 ml of *E. coli* JM109 cultures were harvested and resuspended in 1 ml buffer Z (0.5 M Na_2_HPO_4_, 0.5 M NaH_2_PO_4_, 1 M MgSO_4_), and then 0.1 M of fresh β-mercaptoethanol (Sigma-Aldrich) was added. After permeabilization with 10 µl of toluene, cells were treated with 100 µl of the LacZ substrate ortho-Nitrophenyl-β-galactoside 0.4% (Sigma Aldrich). The reactions were stopped with 120 µl of 1 M Na_2_CO_3_. Measurements were performed as described for PhoA above. Alkaline phosphatase or β-galactosidase activities were normalized to the highest activity obtained in their respective assay.

### Construction of *M. smegmatis* mc^2^155 MS322 and MS321

A 1653 bp fragment of *M. tuberculosis* H37Rv chromosomal DNA, including the full-length *lysX2* gene and 172 bp upstream with its putative promoter, was amplified by PCR with primers Rv1619up and Rv1619low. The PCR-generated fragment was cloned in *E. coli* XL-1 Blue into the *E. coli*–*Mycobacterium* shuttle vector pROLHYG [53] encoding hygromycin resistance, which had been digested with *Bam*HI and HindIII. The resulting plasmid (pROLHYG::Mt_*lysX2*) was propagated in *E. coli* XL-1 Blue after selection on LB agar containing hygromycin 100 µg/ml. The integrity of the coding region was verified by sequencing the inserted DNA fragment. The resulting plasmid pROLHYG::Mt_*lysX2* was electroporated into *M. smegmatis* mc^*2*^155 to obtain MS322. As control, the empty vector pROLHYG was introduced in *M. smegmatis* mc^*2*^155 to obtain the control strain MS321. The expression of *lysX2* in MS322 was analysed by RT-PCR, using primers Rv1619a and Rv1619b (Supplementary Fig. 7, Additional file 1).

### Susceptibility of *M. smegmatis* to HNP-1, HBD-2 and NaNO_2_

*M. smegmatis* susceptibility to HNP-1, HBD-2 and NaNO2 was determined by a resazurin microtiter assay (REMA), as described previously [38]. Briefly, a log-phase bacterial culture grown in 3 ml of Middlebrook 7H9 supplemented with the appropriate antibiotics was diluted to a theoretical OD_540_ of 0.0005 in media without Tween80 and dispensed in a Nunclon 96-well Flat Bottom Black Polystyrol FluoroNunc microplates (Thermo Fisher Scientific) in the presence of serial dilutions of each antimicrobial peptide and sodium nitrite. A growth control containing no antimicrobial peptide and a sterile medium control without bacteria inoculum were also included. Plates were incubated at 37 °C for 48 h and then 10% (v/v) of AlamarBlue (20 µl) (Invitrogen) was added to each well and incubated at 37 °C. Color development was measured after 24 h of incubation using an Infinite 200Pro microplate reader (Tecan Group), with excitation and emission wavelengths of 535 and 590 nm respectively. The lowest drug concentration that resulted in at least 90% inhibition of fluorescence development was considered as the MIC_90_. Experiments were performed in duplicate. Data were reported as mean +/- standard deviation.

### Cell viability in presence of H_2_0_2_

*M. smegmatis* strains MS321 and MS322 were cultured in 10 ml of 7H9 at 37 °C O/N with mild shaking. Subsequently, bacteria were refreshed at OD_600_ of 0.2 corresponding to 2 × 10^− 7^ cfu ml^− 1^. The cultures were then treated with 5 mM of H_2_0_2_ and left in standing for 40 min at 37 °C. The number of viable cells was tested by spreading aliquots of serial dilutions for untreated and treated samples. The survival percentages were calculated by cfu (with H_2_O_2_)/cfu (without H_2_O_2_) for each strain.

### Cell viability at pH 4.5

*M. smegmatis* strains MS321 and MS322 were cultured in 10 ml of 7H9 or Sauton medium at pH 5.5 or pH 6.8 at 37 °C for 24 h with mild shaking. Subsequently, bacteria were centrifuged (3000 rpm, 3 min) and resuspended in the same medium at pH 4.5 with an OD_600_ of 0.01 corresponding to 10^− 6^ cfu ml^− 1^. The number of viable cells was tested by spreading aliquots of serial dilutions at different time points: 0, 4, 8, and 24 h.

### Biofilm cultivation

For biofilm formation, *M. smegmatis* strains were grown in Sauton medium at pH 6.8 or pH 5.5 for 24 h with mild shaking. The cultures were then used to directly inoculate detergent-free Sauton medium, at a 1:100 dilution (OD_600_ ~ 0.01). 4 mL/well of the resulting inoculi were dispensed into 12-well plates, which were then firmly wrapped with parafilm and incubated at 37 °C for up to 7 days. Pellicle formation was documented daily.

### Estimation of Zeta potential

For determination of the bacterial cell surface charge (Bayer & Sloyer, 1990), zeta-potential (f) measurements were performed with 1 OD650 unit PBS-washed single cell suspension in a zetameter Nanosizer ZS90 (Malvern Instruments), at 25 °C.

### Lipid Analysis

*M. smegmatis* MS321 and MS322 were cultured in Sauton medium at pH 5.5 or pH 6.8 at 37 °C for 24 h. After centrifugation, the pellets were treated with a mixture of solvents (CHCl_3_ and CH_3_OH) as previously described [54]. Briefly, two first extractions were performed with 1 volume of CHCl_3_/CH_3_OH (1:2 v/v) followed by 1 volume of CHCl_3_/CH_3_OH (2:1 v/v). The pooled organic extracts were dried under air stream. They were solubilized with CHCl_3_, then transferred in a weighed tube, washed 3 times with distilled water. The organic phase was dried under air stream and weighed. This corresponds to the total lipid extract.

Complex lipids were analyzed by thin layer chromatography (TLC) and lipids were visualized by spraying the plates with appropriate reagents. 250 µg of lipid extracts dissolved in CHCl_3_ were spotted on thin-layer chromatography (TLC) silica gel G Durasil 25 precoated plates (0.25-mm thickness, Macherey-Nagel). For phospholipid analyses, the plates were developed with CHCl_3_/CH_3_OH/H_2_O (60:35:8 v/v/v). After running, the lipids were visualized and characterized with appropriate reagents.

MALDI-TOF-MS experiments were performed on 5800 MALDI TOF/TOF Analyzer (Applied Biosystems/Absciex) equipped with a 349 nm Nd:Yag laser, using the positive ionization and reflection mode by accumulating 40 spectra of 4000 laser shots. Continuous stage motion was selected at a fixed laser intensity of 3500 (instrument-specific units) and 400 Hz pulse rate. Sandwich method was used to deposit the samples on the target plate, spotting 0.5 µl of matrix solution (2,5-dihydroxybenzoic acid (10 mg/ml in CHCl3/CH3OH 1/1, v/v), followed by 0.5 µL of lipid extracts dissolved in chloroform at 1 mM (1 mg/mL) and finally a second droplet of 0.5 µL of matric solution. Deposits were allowed to crystallize at room temperature.

### List of GenBank accession numbers

Sequence identity and similarity have been analyzed by LALIGN (https://fasta.bioch.virginia.edu/fasta_www2/fasta_www.cgi?rm=lalign&pgm=lal). NP_216135.1: MtLysX2; CCP44405.1: MtLysX; AAK58115.1: SaMprF; QHF61974.1: MprF *Listeria monocytogenes*; NP_249611.1: MprF *P. aeruginosa*; AKQ72047.1: MprF *B. licheniformis*; QJF41380.1: MprF *B. subtilis*; EOL94334.1: MprF2 *Enterococcus faecalis*; YP_698880: MprF1 *Clostridium perfringens*; YP_698580: MprF2 *Clostridium perfringens*; AAN52237.1: *Rhizobium tropici*.

### Statistical analysis

Statistical analysis was performed by Student’s t test. Significant levels were indicated as follows: extremely significant: (***); very significant: (**) and significant (*). Non-significant comparisons are not marked in the graphs.

## Supplementary Information

**Additional file 1.**

## Data Availability

All data generated or analysed during this study are included in this published article and its supplementary information files.
