# Supplementary material for: LysX2 is a Mycobacterium tuberculosis membrane protein with an extracytoplasmic MprF-like domain
Source: BMC Microbiol. 2022 Apr 1;22:85. doi: 10.1186/s12866-022-02493-2 (PMC8974105; doi:10.1186/s12866-022-02493-2)
Supplement: Supplementary file 1 — Additional file 1. [file 12866_2022_2493_MOESM1_ESM.pdf]

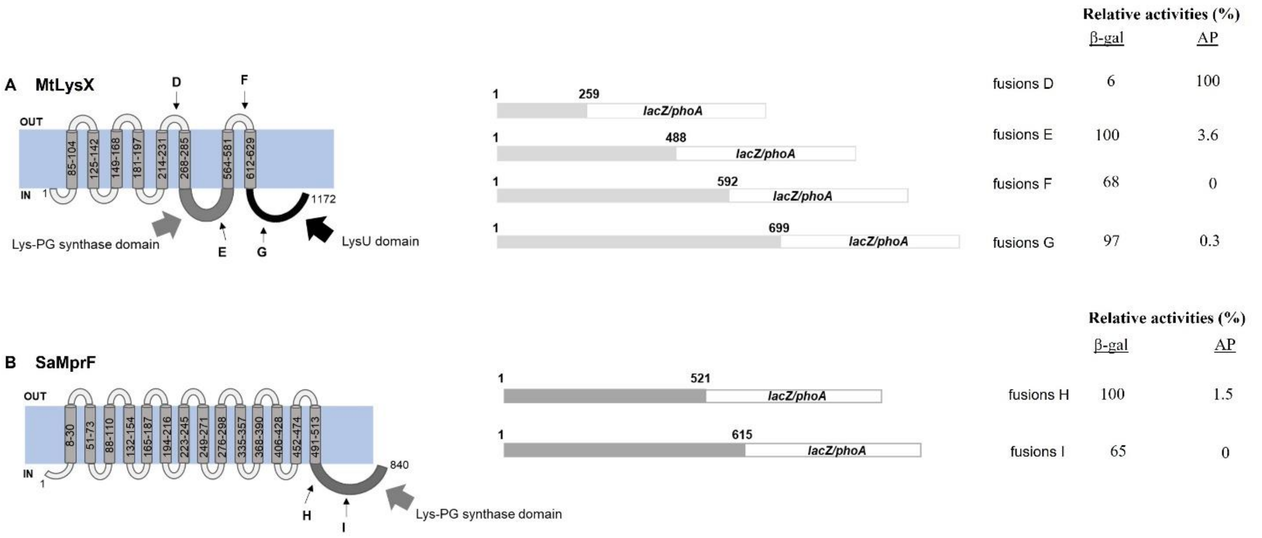

Supplementary Figure 1. Membrane topology of MtLysX and SaMprF. A) Schematic representation of MtLysX (Rv1640c) membrane topology as predicted by HMMTOP and relative activities of the four different fusions of MtLysX (light gray bars) to either *phoA* or *lacZ* (white). β-galactosidase activities (β-gal) were normalized relative to that of fusion E whereas alkaline phosphatase activities (AP) were normalized relative to that of fusion D. The LysU domain of MtLysX [10] is highlighted in black and pointed by a thick black arrow. B) Schematic representation of SaMprF membrane topology as predicted by TMHMM and relative activities of the two different fusions of SaMprF (gray bars) to either *phoA* or *lacZ* (white). β-galactosidase activities (β-gal) were normalized relative to that of fusion H whereas alkaline phosphatase activities (AP) were normalized relative to that of MtLysX fusion D. Relative activities are representative of two independent experiments. The Lys-PG synthase domain of MtLysX and SaMprF is highlighted in dark grey and pointed by a thick gray arrow. IN: inside the cytoplasm. OUT: outside the cytoplasm. The *phoA*/*lacZ* moiety is not drawn on scale as the two genes have a different length. Numbers indicate amino acid positions.

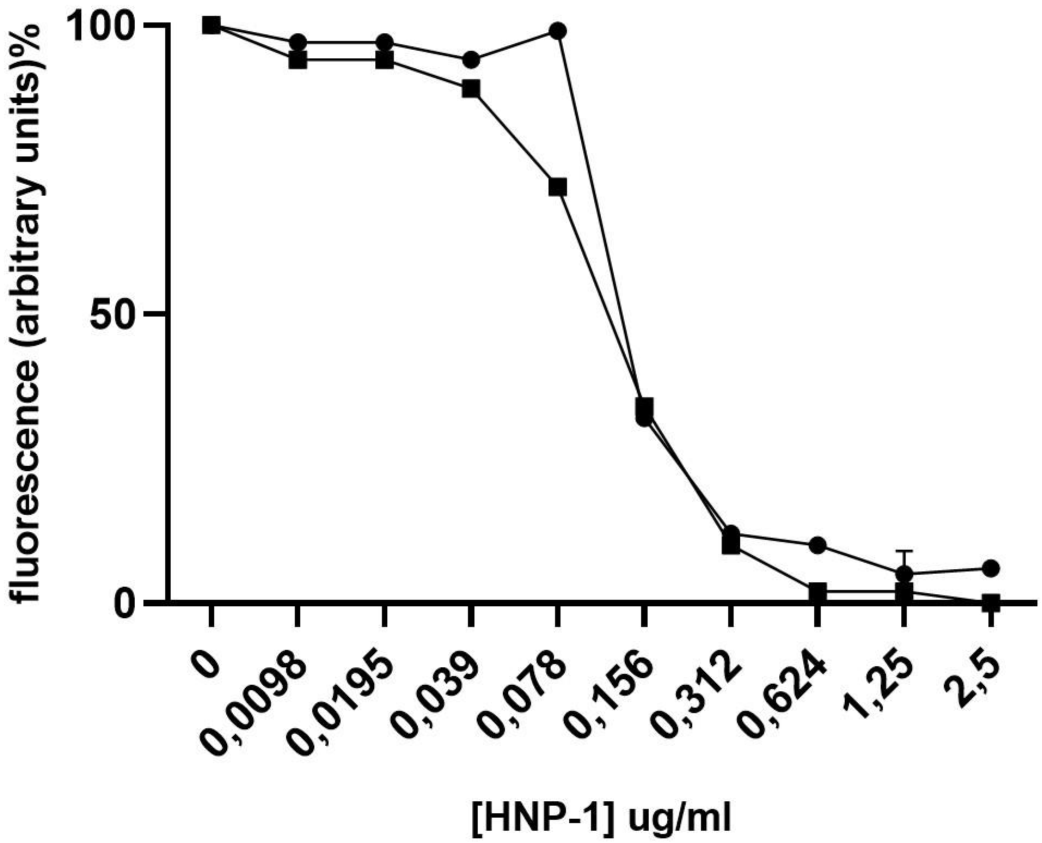

Supplementary Figure 2. Minimum inhibitory concentration of HNP-1 in *M. smegmatis* expressing MtLysX2 (MS322, dotted line) compared to the parental strain (MS321, squared line)**.** Error bars derive from two independent experiments.

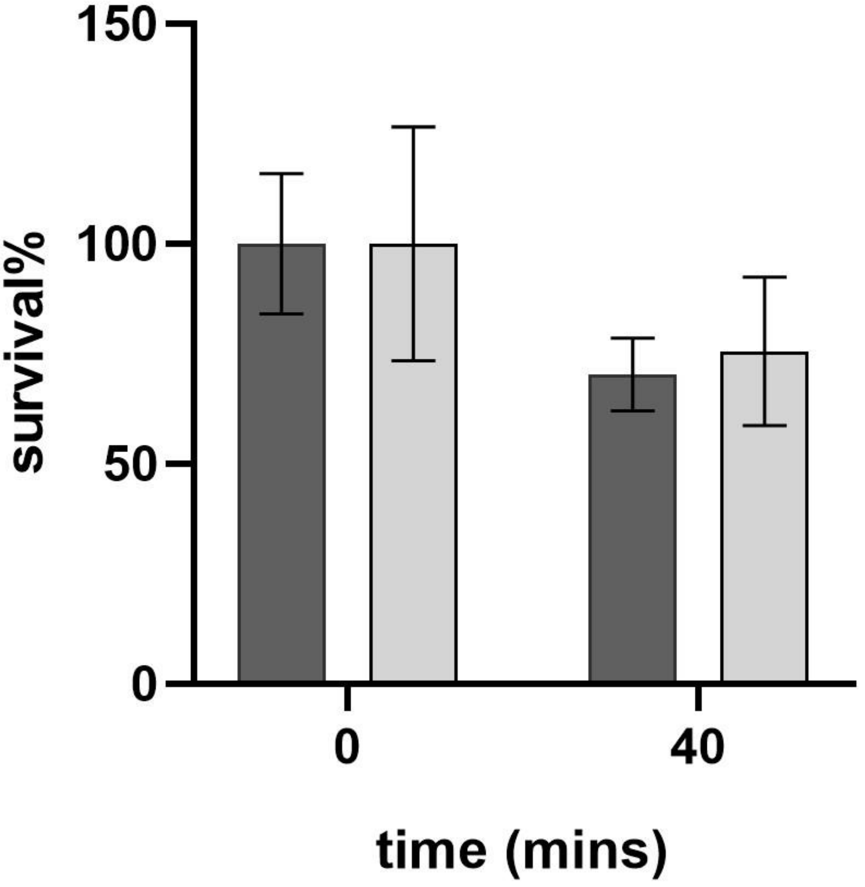

Supplementary Figure 3. Killing assay with hydrogen peroxide. Killing assay of *M. smegmatis* mc^{2}155::Mt_*lysX2* (MS322, dark gray) and mc^{2}155::pROL_Hyg (MS321 light gray) with 5mM $H_2 0_2$.

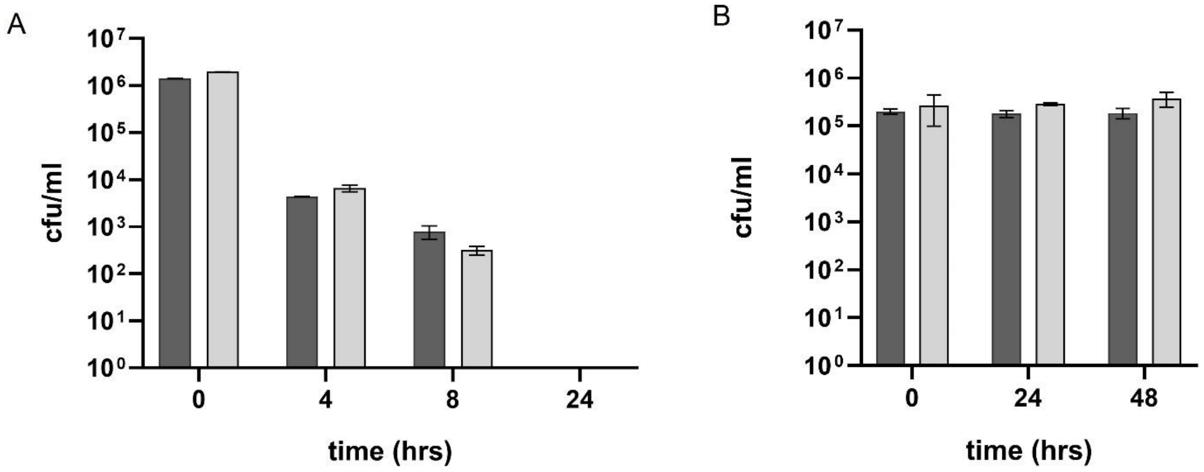

Supplementary Figure 4. Killing assay of *M. smegmatis* mc^{2}155::*Mt_lysX2* (MS322, dark gray) and mc^{2}155::pROL_Hyg (light gray) at pH 4.5. Strains were grown for 24 h in Sauton medium at pH 6.8 (A), and 7H9 at pH 5.5 (B) before being transferred into the same medium at pH 4.5.

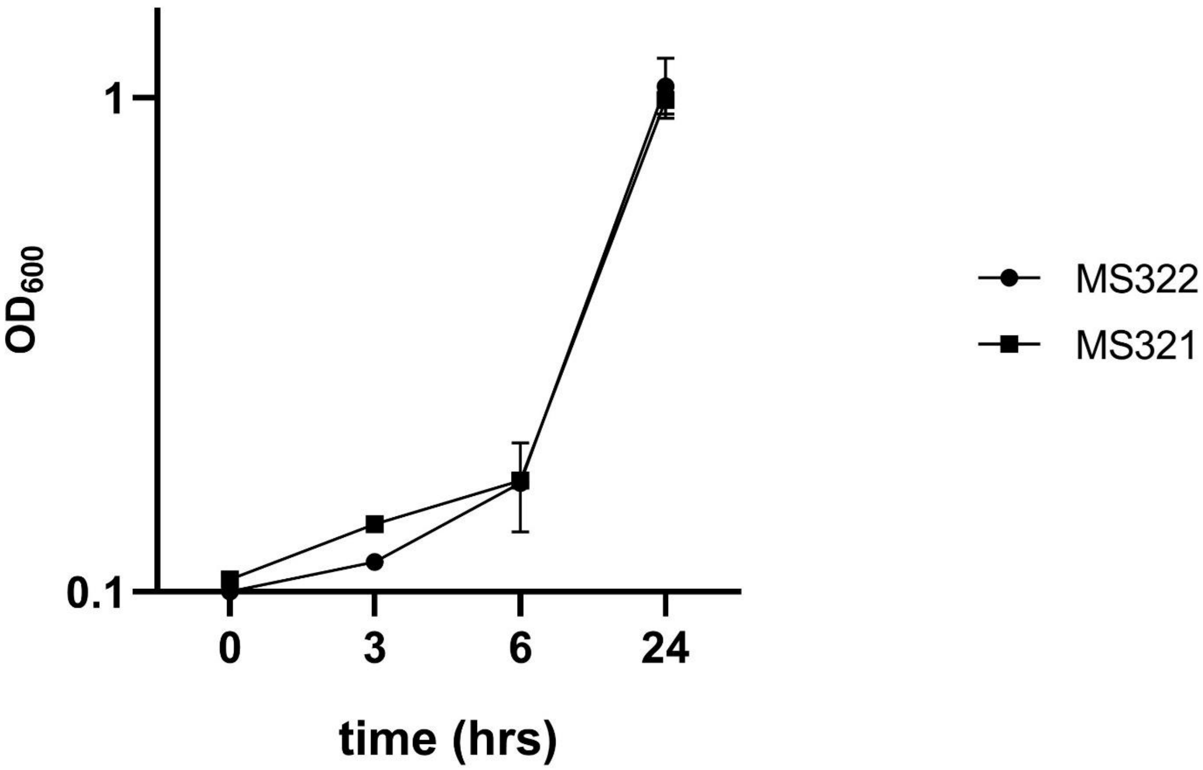

Supplementary Figure 5. Growth curve of MS321 and MS322 in Sauton medium pH 5.5. The growth curve is representative of two independent experiments.

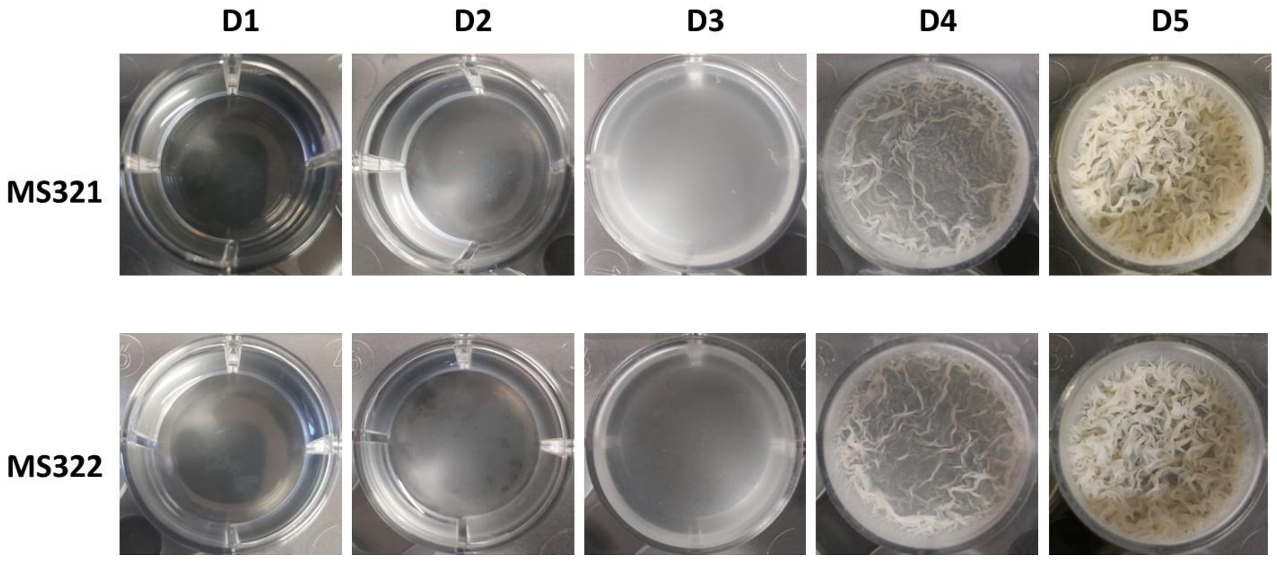

Supplementary Figure 6. Biofilm formation in Sauton medium pH 6.8. Bacteria, grown to stationary phase were diluted 1:100 in Sauton pH 6.8. 4 ml of the suspension were than inoculated in a 24-well plate and

incubated at 37°C. Pictures were taken after 1, 2, 3, 4 and 5 days (D). The experiment was repeated twice in triplicate and comparable results were obtained.

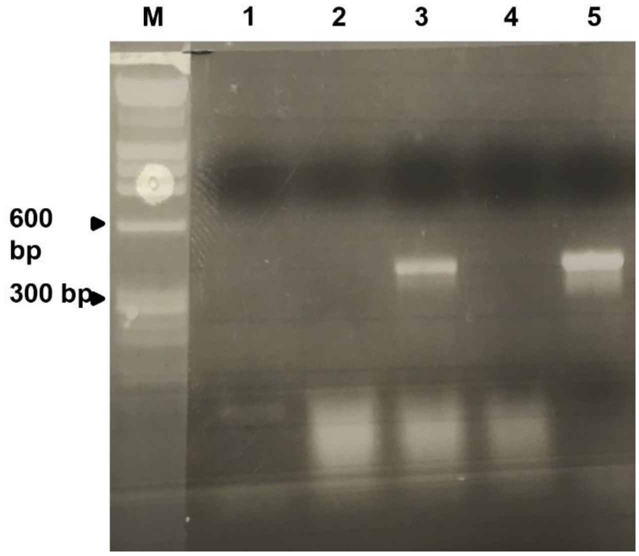

Supplementary Figure 7. Expression of *MtlysX2* in *M. smegmatis*. The image shows an ethidium-bromide-stained gel of the RT-PCR products, separated by 2% agarose gel electrophoresis. M, molecular mass marker; 1, H_2O; 2, RNA from MS322; 3, cDNA from MS322; 4, cDNA from MS321; 5, DNA from *M. tuberculosis* H37Rv.

Supplementary Table S1. List of primers. Sequences recognized by the restriction enzymes used in this work are underlined. RBS is highlighted in italics. Sequences annealing to *M. tuberculosis* are highlighted in bold.

Mt_lysX2::phoA

Primer name	Sequence	Primer orientation	Restriction enzymes	PCR fragment
RP578	GAGCTCTTAA*GAAGGA*GATATACAT**ATGGTGGCAGCGGCCGGCGAGC**	forward	SacI, NdeI	fusion A
RP582	CAT**ATGGTGGCAGCGGCCGGCGAGC**	forward	NdeI	fusion B and C
RP583	GTCGACA**GGCCGCGGTCGCATGCATGGC**	reverse	SalI	fusion A
RP585	GTCGACA**GTGACACATGGCCGCGAAGTCG**	reverse	SalI	fusion B
RP587	GTCGACA**GATCAACGCACGGCAGACACGCT**	reverse	SalI	fusion C

Mt_lysX2::lacZ

Primer name	Sequence	Primer orientation	Restriction enzymes	PCR fragment
RP580	GAATTCTTAA*GAAGGA*GATATACAT**ATGGTGGCAGCGGCCGGCGAGC**	forward	EcoRI, NdeI	fusion A
RP1454	TATACAT**ATGGTGGCAGCGGCCGGCGAGC**	forward	NdeI	fusion B and C
RP584	GGATCCTT**GGCCGCGGTCGCATGCATGGC**	reverse	BamHI	fusion A
RP586	GGATCCAC**GTGACACATGGCCGCGAAGTCG**	reverse	BamHI	fusion B
RP588	GGATCCAA**GATCAACGCACGGCAGACACGCT**	reverse	BamHI	fusion C

Mt_lysX::phoA

Primer name	Sequence	Primer orientation	Restriction enzymes	PCR fragment
RP1508	GAGCTCTTAA*GAAGGA*GATATACATA**TGGGACTCCACTTAACTGTCCCTGGCCTTC**	forward	SacI, NdeI	fusion D
RP1510	CATA**TGGGACTCCACTTAACTGTCCCTGGCCTT**	forward	NdeI	fusion E, F, and G
RP1509	TCTAGAT**GGGATCGGCGAGGGCGAATC**	reverse	XbaI	fusion D
RP1511	TCTAGA**CCACCAGCAGGCAGTCGGAGTC**	reverse	XbaI	fusion E
RP1512	TCTAGA**TGTTGGAGCGGTAGAGCGTCTCGA**	reverse	XbaI	fusion F
RP1513	TCTAGAT**TACTTGCCGCAGCCCGCTCACGTC**	reverse	XbaI	fusion G

Mt_lysX::lacZ

Primer name	Sequence	Primer orientation	Restriction enzymes	PCR fragment
RP1514	GATATCTTAA*GAAGGA*GATATACATATGGGACTCCACTTAACTGTCCCTGGCCTTC	forward	EcoRV, NdeI	fusion D
RP1510	CATA**TGGGACTCCACTTAACTGTCCCTGGCCTT**	forward	NdeI	fusion E, F, and G
RP1515	GGATCCTT**GGGATC GGCGAGGGCGAATC**	reverse	BamHI	fusion D
RP1516	GGATCC**TCCACCAGCAGGCAGTCGGAGTC**	reverse	BamHI	fusion E
RP1517	GGATCCT**TGTTGGAGAGGTAGAGCGTCTCGA**	reverse	BamHI	fusion F
RP1518	GGATCCTT**TACTTGCCGCAGCCCGCTCACGTC**	reverse	BamHI	fusion G

Sa mprF::phoA

Primer name	Sequence	Primer orientation	Restriction enzymes	PCR fragment
RP1581	AACAT**ATG****AATCAGGAAGTTAAAAACAAA**	forward	SacI	fusion H and I
RP1582	AAGGATCC**TAGAAGAAATACGTACTTTGCTAA**	reverse	BamHI	fusion H
RP1583	AAGGATCC**CGAAATTATGATATAAAGGCATGT**	reverse	BamHI	fusion I

Sa mprF::lacZ

Primer name	Sequence	Primer orientation	Restriction enzymes	PCR fragment
RP1578	AACAT**ATGAATCAGGAAGTTAAAAACAAA**	forward	EcoRI	fusion H and I
RP1579	AAGGATCC**TTAGAAGAAATACGTACTTTGCTA**	reverse	BamHI	fusion H
RP1580	AAGGATCC**CCGAAATTATGATATAAAGGCAT**	reverse	BamHI	fusion I

M. smegmatis::Mt_lysX2

Primer name	Sequence	Primer orientation	Restriction enzymes	PCR fragment
Rv1619up	GTTGGGATCCGGGCACTTT	forward	BamHI	*lysX2*+172 bp upstream
Rv1619low	TGCGCCAAGCTTTCCATAG	reverse	HindIII	*lysX2*+172 bp upstream
Rv1619a	GTGACTGTGAAGCTGC	forward		*lysX2* 355bp fragmen*t*
Rv1619b	CGGCTATCAGAATCTCACC	reverse		*lysX2* 355bp fragment
